# A prospective study revealing the role of an immune-related eRNA, WAKMAR2, in breast cancer

**DOI:** 10.1038/s41598-021-94784-3

**Published:** 2021-07-28

**Authors:** Linbang Wang, Jingkun Liu, Jiaojiao Tai, Nian Zhou, Tianji Huang, Yuzhou Xue, Zhengxue Quan

**Affiliations:** 1 Department of Orthopedic Surgery, The First Affiliated Hospital of Chongqing Medical University, Chongqing, 400016 China; 2 Honghui Hospital, Xian Jiaotong University, Xi’an, 710072 China; 3 Department of Cardiology, The First Affiliated Hospital of Chongqing Medical University, Chongqing, 400016 China

**Keywords:** Cancer microenvironment, Breast cancer, Tumour biomarkers

## Abstract

Enhancer RNAs (eRNAs) are a subclass of non-coding RNAs that are generated during the transcription of enhancer regions and play an important role in tumourigenesis. In this study, we focused on the crucial eRNAs that participate in immune responses in invasive breast cancer (IBC). We first used The Cancer Genome Atlas and Human enhancer RNA Atlas to screen for tissue-specific eRNAs and their target genes. Through Pearson correlation analysis with immune genes, the eRNA WAKMAR2 was identified as a key candidate involved in IBC. Our further research suggested that WAKMAR2 is crucial in regulating the tumour microenvironment and may function by regulating immune-related genes, including IL27RA, RAC2, FABP7, IGLV1-51, IGHA1, and IGHD. Quantitative reverse transcription-polymerase chain reaction was used to detect the expression of WAKMAR2 in IBC and normal tissues, and the effect of WAKMAR2 on the regulation of downstream genes in MB-231 and MCF7 cells was studied in vitro. WAKMAR2 was found to be highly involved in tumour immunity and was downregulated in IBC tissues. Furthermore, the expression of WAKMAR2 and its target genes was observed at the pan-cancer level. This study provides evidence to suggest new potential targets for the treatment of breast cancer.

## Introduction

Female breast cancer (BC) is the most commonly diagnosed cancer across the globe^1^ and it has been recognised as a biologically heterogeneous disease with different aetiologies and responses to treatments^2^. Previous studies have shown that multiple factors, including genetic susceptibility, are associated with a high risk of BC^3^. However, the contributions and mechanisms of these risk factors to the biogenesis and progression of BC are poorly understood^4^.

In addition to general surgical treatment and neoadjuvant chemotherapy, other treatments for BC include endocrine therapy^5^ and immunotherapy^6^. Recent studies have found that 22 genes can be used as targets for BC immunotherapy^7^, and long non-coding RNA T-cell leukaemia/lymphoma 6 (TCL6) may play a protective role in BC through immune infiltration^8^.

Enhancers are cis-acting DNA sequences that can increase the rate of gene transcription^9^. Most enhancers with epigenetic characteristics are transcribed into non-coding RNAs, which include the class enhancer RNAs (eRNAs)^10^. There is increasing evidence that eRNAs play a key role in tumour development and treatment^11–13^. Zhang et al.^14^ and Lee et al.^11^ suggested that eRNAs can be used as a new direction for the treatment of human cancer. Gain-of-function and loss-of-function experiments showed that heparanase (HPSE) eRNA can promote tumour growth and invasion in vivo and in vitro^15^. Inhibition of antisense eRNA has a protective effect against prostate cancer^13^.

In this study, we first screened eRNAs related to the survival of breast cancer patients and then selected immune-related genes as targets for constructing an eRNA-immune gene regulatory network. WAKMAR2, which had the highest node degree, was identified as the key eRNA. Molecular biological methods were used to determine the expression of WAKMAR2 in patients with invasive breast cancer (IBC). WAKMAR2 was highly expressed in the paracancerous tissues of IBC patients and hyperimmune patients. Finally, bioinformatic analysis was used to predict the potential function of WAKMAR2 in IBC through its downstream targets. In summary, we identified WAKMAR2 as a potential molecular marker for the prognosis of patients with IBC.

## Results

### Study design and data processing

**Figure 1:**
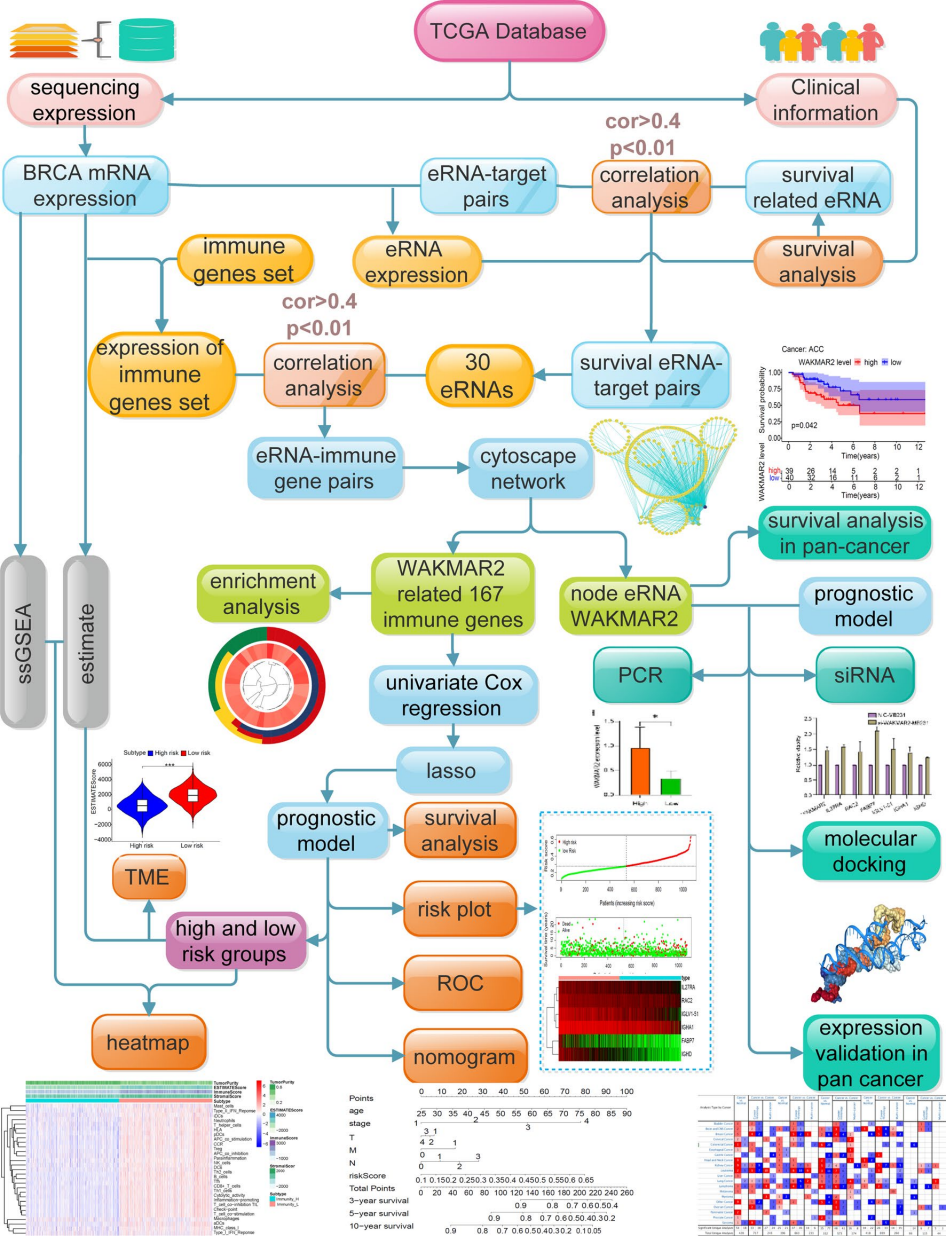
The overall study design process.

As shown in Fig. 1, key eRNAs and their potential targets in IBC were screened by Kaplan–Meier survival analysis and Pearson correlation analysis, while the network of potential eRNA-immune target pairs was constructed using Cytoscape. Further enrichment analysis revealed that the eRNA WAKMAR2 may play a crucial role in tumour immunity. Next, the least absolute shrinkage and selection operator (LASSO) method was applied to screen for the target genes of WAKMAR2, and a prognosis-related model with six target genes was constructed. PCR and in vitro experiments using siRNA were used to validate the bioinformatic results.

### Prognostic eRNAs and their targets in IBC

**Figure 2:**
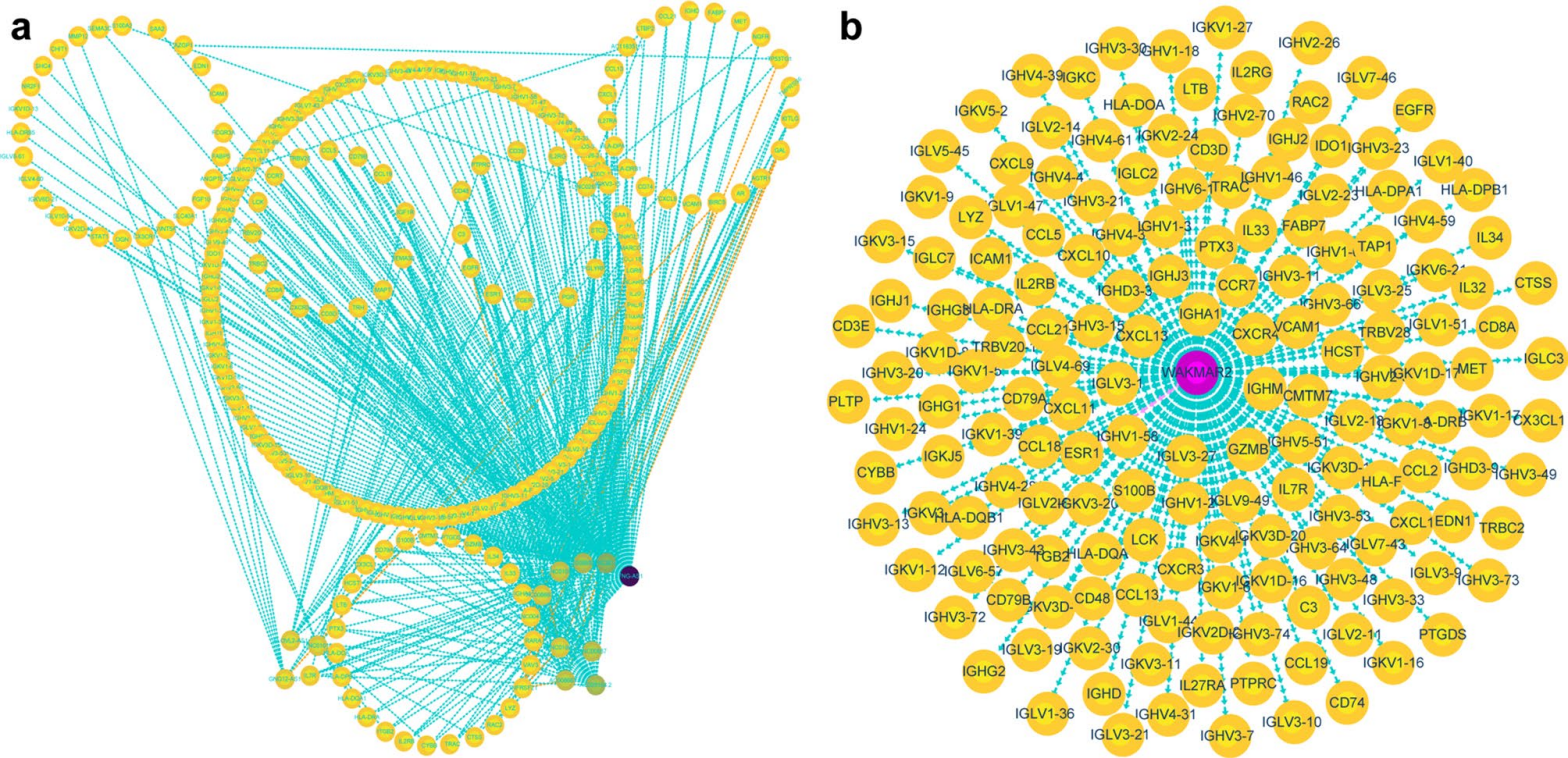
Interaction network between enhancer RNA (eRNA) and immune genes in invasive breast cancer. (**a**) Overall interaction network. Each circle represents an eRNA or a gene, and the darker the colour, the higher the node. The connection line represents the interaction between eRNA and immune genes, the blue line indicates a positive correlation, and the orange line indicates a negative correlation. (**b**) WAKMAR2 and its related target gene interaction network. Blue lines indicate a positive correlation, and pink-purple lines indicate a negative correlation.

**Figure 3:**
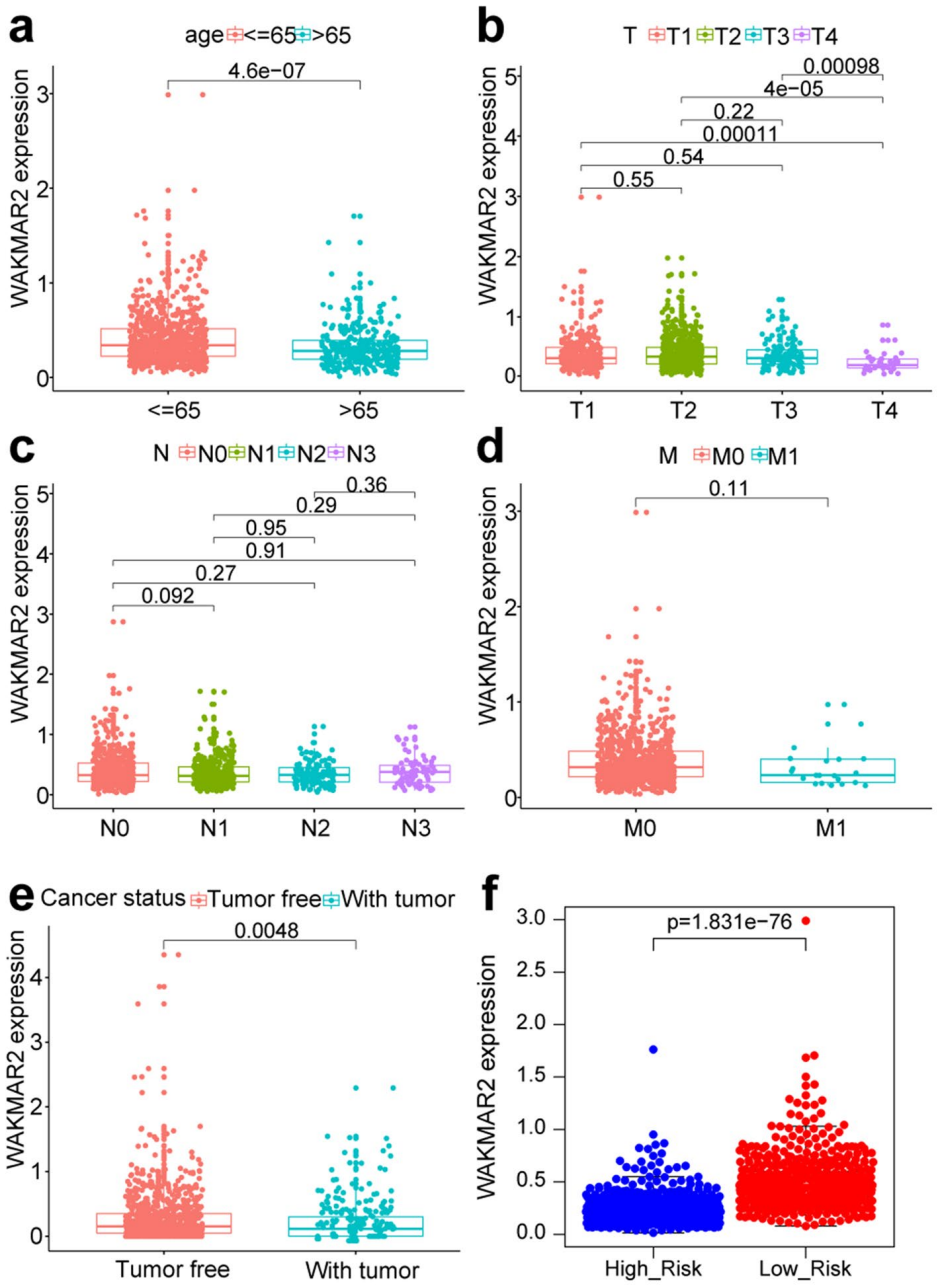
Relationship between WAKMAR2 expression and clinical characteristics, in detail, N represents the degree of spread to regional lymph nodes, including N0: no regional lymph nodes metastasis, N1: regional lymph node metastasis present; at some sites, tumor spread to closest or small number of regional lymph nodes, N2: tumor spread to an extent between N1 and N3 (N2 is not used at all sites), and N3: tumor spread to more distant or numerous regional lymph nodes; T represents the size or direct extent of the primary tumor, in which the T1, T2, T3, T4 stands for the size and extension of the primary tumor; M represents the presence of distant metastasis, including M0: no distant metastasis, and M1: metastasis to distant organs (beyond regional lymph nodes).

**Figure 4:**
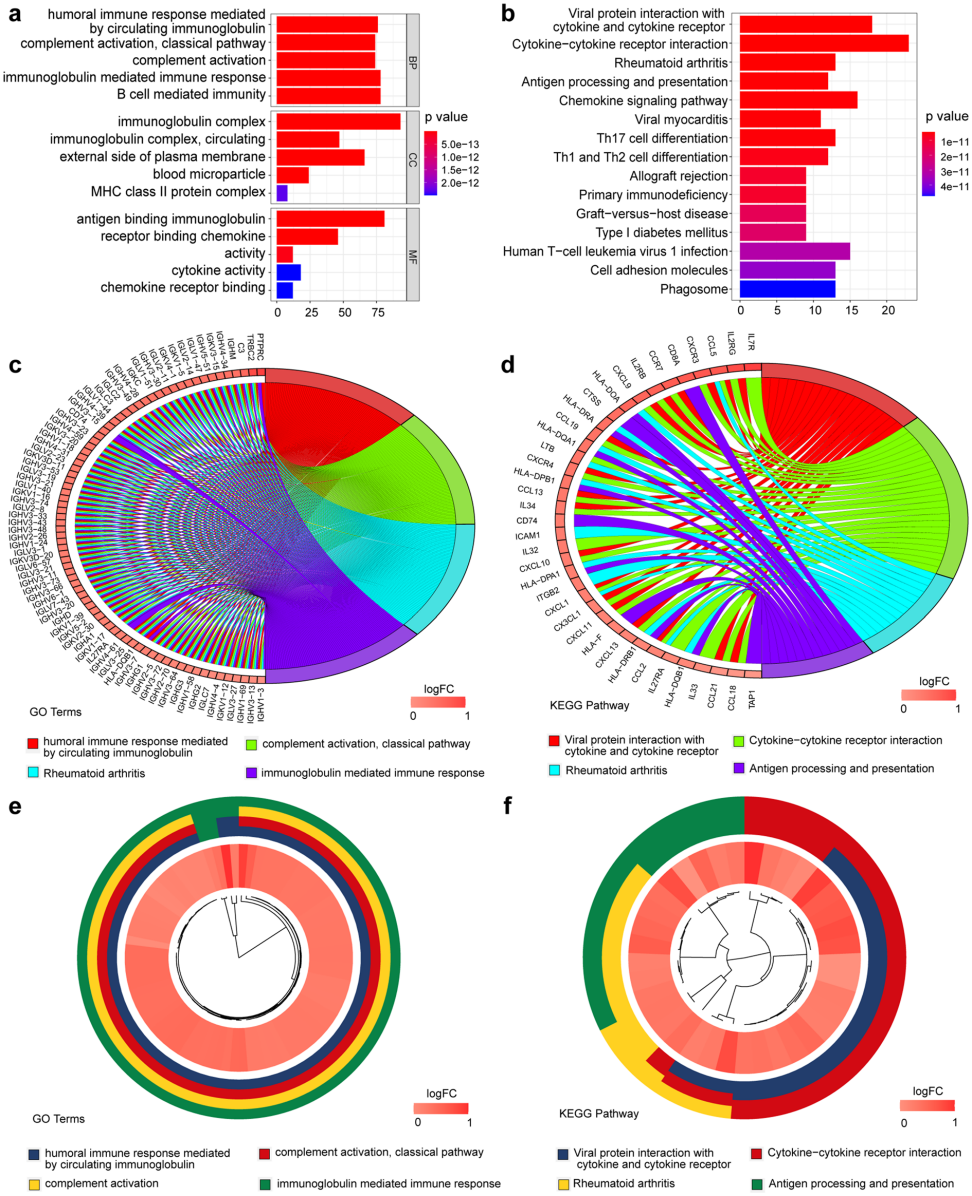
Enrichment analysis of WAKMAR2-related immune genes.

A total of 91 survival-related eRNAs were identified among 1580 eRNAs, of which 30 had a correlation coefficient greater than 0.4 for the target gene obtained from the Human enhancer RNA Atlas (HeRA) website. Following correlation analysis between these 30 eRNAs and 1811 immune genes, 18 eRNAs and 226 immune genes were obtained. Figure 2a shows the regulatory network of 18 eRNAs and 226 immune genes, while Fig. 2b shows the regulatory network of WAKMAR2 and 167 downstream genes. Clinical correlation analysis showed that the expression of WAKMAR2 was associated with clinical features such as tumour status, patient age at diagnosis, and T stage (Fig. 3a, b, e) but not associated with N stage and M stage(Fig. 3c, d). The results of Gene Ontology (GO) and Kyoto Encyclopedia of Genes and Genomes (KEGG) enrichment analyses, shown in Fig. 4, suggest that WAKMAR2 may play a role in IBC through several mechanisms such as cytokine activity, the MHC class II protein complex, and immunoglobulin-mediated immune responses.

### WAKMAR2 regulates downstream target genes in IBC

**Figure 5:**
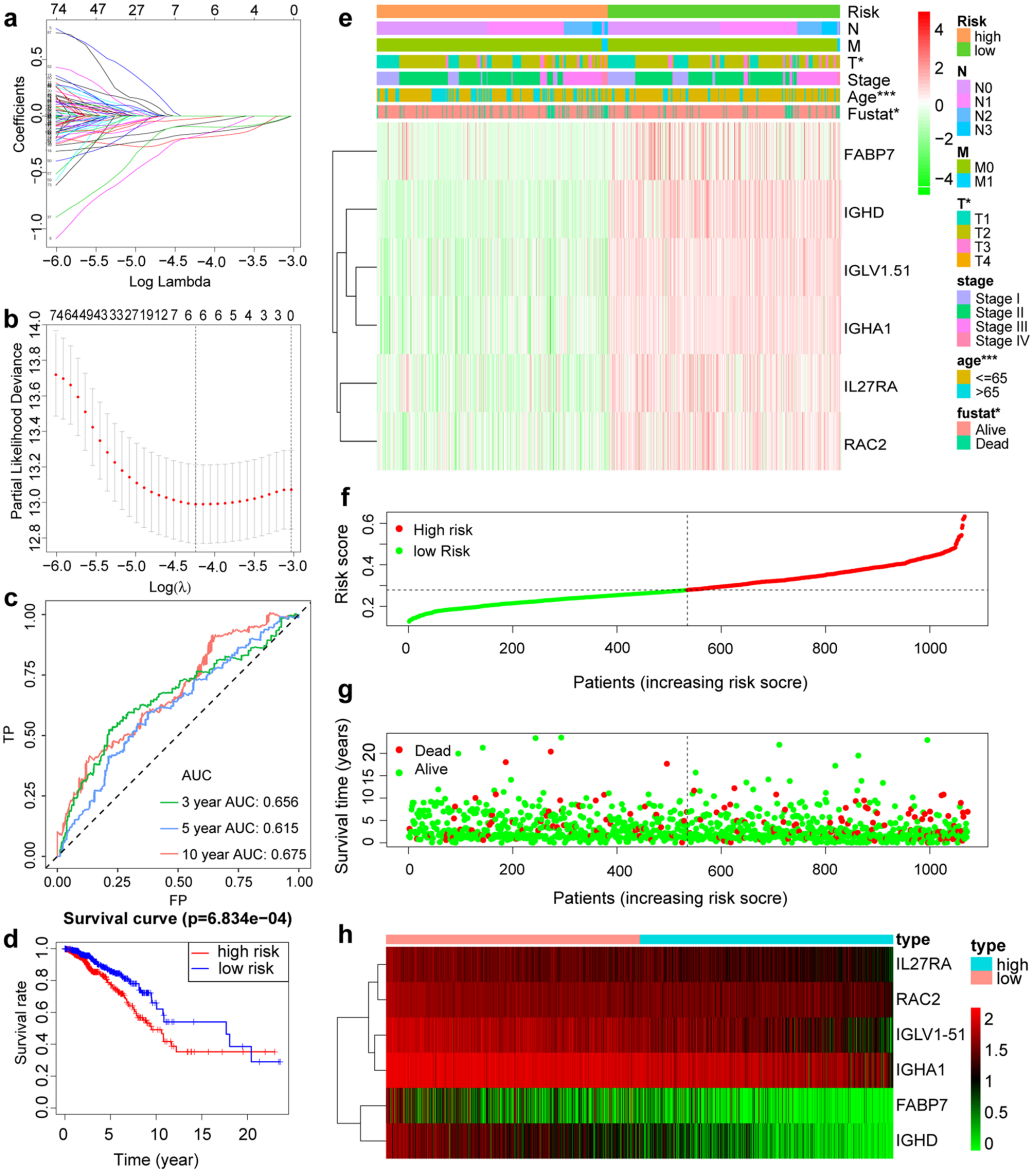
Construction and verification of a prognostic model. (**a**, **b**) Construction of a prognostic model via LASSO cox regression analysis. (**c**) Time-dependent ROC analysis of a prognostic model. (**d**) Kaplan–Meier analysis of patients with high and low risk. (**e**) Heatmap of the correlation between clinical features and a prognostic model. (**f**–**h**) Risk distribution curve (**f**), survival status (**g**), the six prognostic gene expression heatmap (**h**) distribution in the high- and low-risk groups.

**Figure 6:**
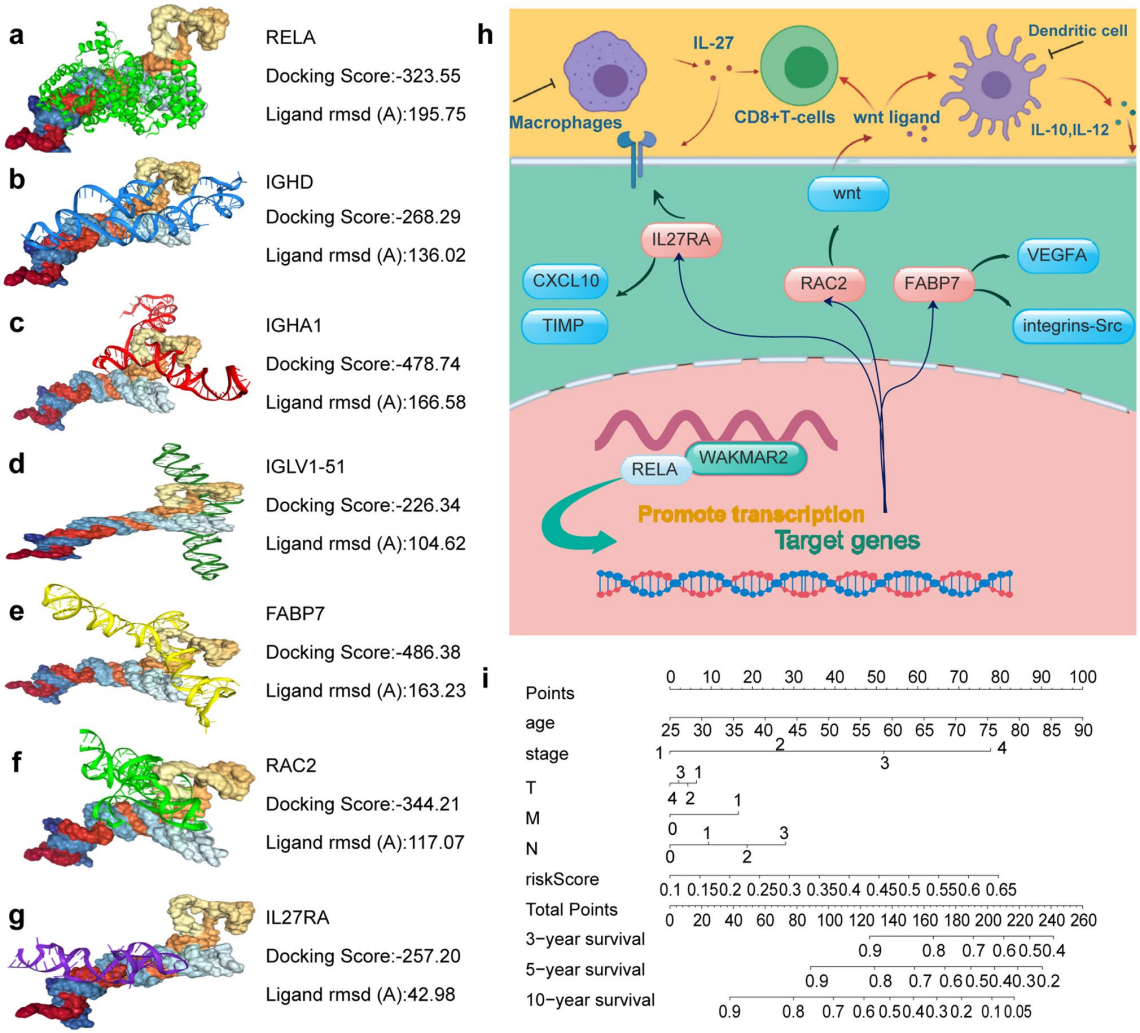
Molecular docking, schematic diagram, and nomogram of WAKMAR2. (**a–g**) Molecular docking of WAKMAR2 with transcription factors and immune prognostic target genes. (**h**) Schematic diagram of WAKMAR2 action mechanism. (**i**) A prognostic nomogram predicting 3-, 5-, and 10-year overall survival with breast cancer. () ChIP-qPCR analysis of RELA binding to the according region of FABP7, IGHA1, RAC2 in MB231 cells transfected with control or si-WAKMAR2. Immunoprecipitated DNAs were detected by real-time PCR.

First, Ensembl was applied to further confirm that WAKMAR2 belongs to the eRNA (http://asia.ensembl.org/Homo_sapiens/Location/View?db=core;g=ENSG00000237499; *r* = 6:137822782–137869124), as shown in sspplementary Fig. 1, WAKMAR2 is located in the enhancer region^16^. Next, in order to determine the specific targets of WAKMAR2 in IBC immune regulation, a univariate Cox regression analysis was used to screen 121 immune genes, of which six genes, IL27RA, RAC2, FABP7, IGLV1-51, IGHA1, and IGHD, were included in the subsequent LASSO analysis prognostic model (Fig. 5a, c). The risk score for each patient was calculated according to the linear combination of the expression levels of each gene-weighted risk coefficient. Kaplan–Meier survival curve analysis revealed that the survival rate of patients in the low-risk group was higher than that in the higher-risk group (Fig. 5g). Receiver operating characteristic (ROC) curve analysis showed that the area under the curve (AUC) for 3, 5, and 10 years was 0.656, 0.615, and 0.675, respectively, which proved that our model had reliable stability (Fig. 5e). A nomogram was set up to assist clinicians in quantifying the prognosis of patients with IBC (Fig. 6i). As shown in Fig. 5b, h, the heat map revealed that IL27RA, RAC2, FABP7, IGLV1-51, IGHA1, and IGHD were highly expressed in low-risk patients (Fig. 5e). The risk curve (Fig. 5d) and scatter plot (Fig. 5f) showed the impact of these six genes on the overall cohort survival. A high risk value for IBC patients was associated with a high mortality rate, which was consistent with the results of Kaplan–Meier analysis, also, the expression of WAKMAR2 was associated with risk status in different group (Fig. 3f).

### WAKMAR2 is crucial for immune regulation in IBC

**Figure 7:**
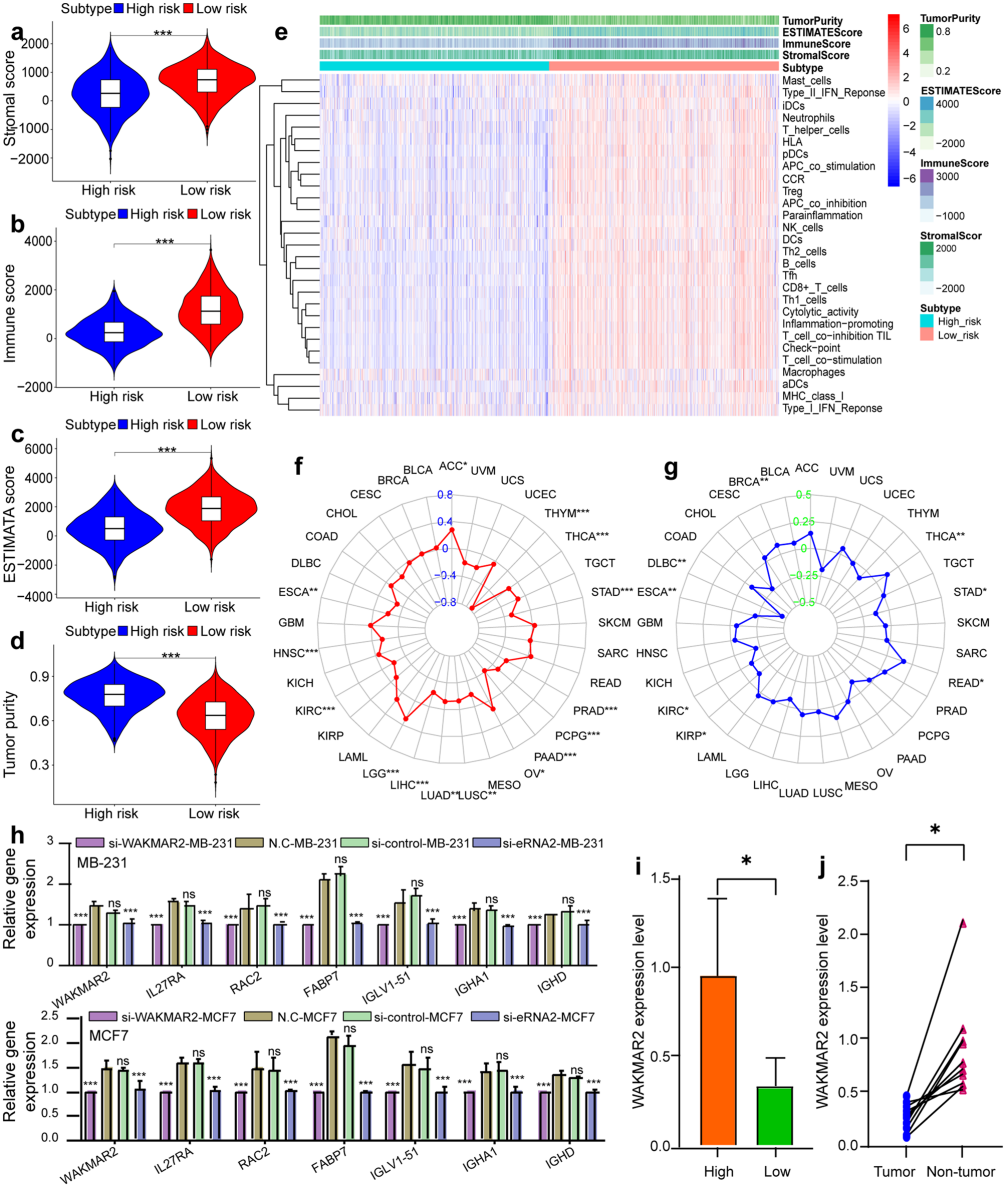
WAKMAR2 expression with tumour microenvironment (TME) relevance for invasive breast cancer (IBC) patients. IBC samples were divided into high-risk (blue band) and low-risk clusters (red band). The assessment of infiltration clusters in the TME was clustered into a stromal score (**a**), an immune score (**b**), an estimate score (**c**), and tumour purity (**d**). (**e**) ssGSEA results show the detailed immune infiltration landscapes of different clusters. Correlation between WAKMAR2 expression and tumour mutation burden (**f**) and microsatellite instability (**g**) in pan-cancer (**h**). Expression levels of WAKMAR2 and its targets after WAKMAR2 knockdown. (**i**) WAKMAR2 expression in higher and lower immunity groups, where “High” stands for the higher immunity group and “Low” stands for the lower immunity group. (**j**) WAKMAR2 expression in IBC and normal tissues.

A scatter plot showed that the expression of WAKMAR2 in the high-risk group was significantly lower than that in the low-risk group (Fig. 3f), indicating that it has a protective effect on the occurrence and development of IBC. Figure 7a–d shows that the low-risk group had higher stromal and immune scores, while the high-risk group had higher tumour purity, indicating increased immune cell infiltrate in the low-risk group. The 29 immune cell subtypes, including B cells, CD8 + T cells, NK cells, neutrophils, macrophages, and mast cells, were highly expressed in the low-risk group (Fig. 7e), which was consistent with the results from tumour microenvironment (TME) studies. Furthermore, in order to detect the relationship between WAKMAR2 expression and the immune cell fractions, we divided all samples into WAKMAR2 high-expression and low-expression group, the relative fractions of immune cell subpopulations in different clusters inferred by Xcell were consisted with our predictions.

The results of molecular docking between WAKMAR2 and its target genes, shown in Fig. 6, suggest that they have a stable binding affinity. Next, the potential transcription factors (TFs) that WAKMAR2 recruits to transregulate the target gene were identified, and RELA was screened as a hub TF with the highest docking score (Fig. 6a–g). Furthermore, ChIP-PCR experiment confirmed that WAKMAR2 effected the RELA binding to the according region of FABP7, IGHA1, and RAC2 (Fig. 7e). To examine the role of WAKMAR2 in regulating target gene expression, WAKMAR2 was knocked down with siRNA in MB-231 and MCF7 cells, the knock-down siRNA efficiency percentage of WAKMAR2 are listed as below: the efficiency of siRNA1 in MD-231 is 29.51% and 29.82% in MCF7; the efficiency of siRNA2 in MD-231 is 30.61%, and 34.20% in MCF7; the mRNA level expression of IL27RA, RAC2, FABP7, IGLV1-51, IGHA1, and IGHD was found to be downregulated and the protein level of FABP7 and IGHA1 was found to be downregulated in the knockdown cells (Fig. 7h). Finally, we performed qRT-PCR on the cancer samples collected from patients with triple-negative breast cancer. Compared with that in para-carcinoma tissue, the expression of WAKMAR2 in tumour tissue was significantly reduced. Furthermore, WAKMAR2 expression varied with patient immune scores (Fig. 7i,j).

### Pan-cancer analysis

**Figure 8:**
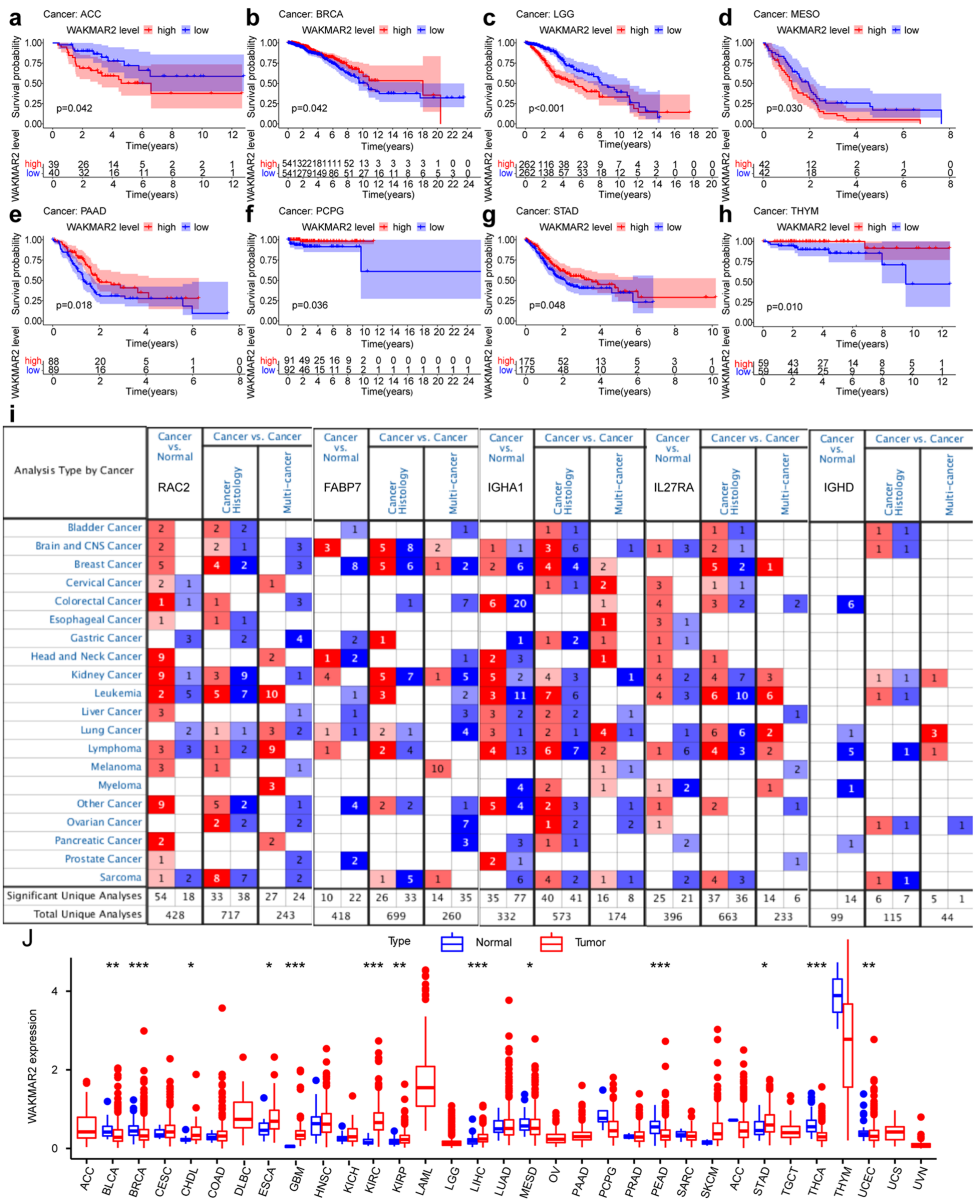
Pan-cancer verification. (**a**–**h**) Survival analysis of WAKMAR2 in pan-cancer. (**i**) The oncomine database results verified the expression of WAKMAR2 target prognostic genes in pan-cancer. (**j**) Differential expression verification of WAKMAR2 in pan-cancer.

Pan-cancer analysis of WAKMAR2 is shown in Fig. 8j. Kaplan–Meier survival analysis of pan-cancer results showed that patients with higher WAKMAR2 expression in adrenocortical carcinoma (ACC), brain lower grade glioma (LGG), and mesothelioma (MESO) had a markedly shorter overall survival than those with low WAKMAR2 expression. Patients with higher WAKMAR2 expression had significantly longer overall survival than the patients whose WAKMAR2 expression was lower, such as in breast invasive carcinoma (BRCA), pancreatic adenocarcinoma (PAAD), Pheochromocytoma/paraganglioma (PCPG), stomach adenocarcinoma (STAD), and thyoma (THYM) (Fig. 8a–h). The tumour mutational burden (TMB) level is associated with sensitivity to inhibitors of immune checkpoint^17,18^. We found that the expression of WAKMAR2 in other cancers, including ACC, THCA (Thyroid Cancer), STAD (stomach adenocarcinoma), PRAD (prostate adenocarcinoma), PCPG (paraganglioma), PAAD (Pancreatic adenocarcinoma), OV (Ovarian carcinoma), LUSC (Lung squamous carcinoma), LUAD (lung adenocarcinoma), LIHC (hepatocellular carcinoma), LGG (low-grade glioma), KIRC (Kidney renal clear cell carcinoma), HNSC (head and neck squamous cell carcinoma), and ESCA (Esophageal carcinoma), was correlated with TMB, while in BRCA (Breast Invasive Carcinoma), THCA (thyroid carcinoma), STAD (stomach adenocarcinoma), READ (rectum adenocarcinoma), KIRC (Kidney renal clear cell carcinoma), ESCA (Esophageal carcinoma), and DLBC (diffuse large B cell lymphoma), its expression was correlated with microsatellite instability (MSI) (Fig. 7f, g). Finally, using the Oncomine database, we compared the transcription levels of prognostic target genes in cancer and normal samples at the pan-cancer level (Fig. 8i).

## Discussion

For many years, eRNAs have been recognised as a by-product of the transcription of DNA enhancers^19^. The critical function of eRNAs in the control of gene transcription in tumour cells has been recognised. They play a role in multiple crucial signalling pathways by regulating target genes^14^ through both cis- and trans-regulatory activities^20^. Here, we identified WAKMAR2 as a crucial eRNA in the IBC tumour immune system.

Significant heterogeneity has been found in the tumour microenvironment of patients with IBC and this has been shown to result in varying immunotherapeutic effects^21^. In the present study, Pearson correlation analysis between eRNA and immune genes was performed, interaction networks were constructed using Cytoscape, and the hub eRNA WAKMAR2 was screened out. WAKMAR2 has been recognised as a crucial regulator in skin wound healing, and it is also a transcript encoded by RNA polymerase II. High expression of WAKMAR2 is induced by TGF-β signalling in keratinocytes, thereby restricting their production of inflammatory chemokines and enhancing cell migration^22^. However, the biological significance of WAKMAR2 in downregulation remains unclear. Enrichment analysis of WAKMAR2 target genes revealed several crucial pathways, including those involved in cytokine activity, MHC class II protein complexes, and immunoglobulin-mediated immune responses, that are potentially regulated by WAKMAR2. Cytokines can increase the number of immune cells in the tumour microenvironment and play a critical role in tumourigenesis^23^. MHC class II is a classic gene that controls specific immune responses and aids in the delivery of exogenous peptide antigens to CD4 + T cells, thus promoting a humoral immune response^24^. Therefore, we can speculate that WAKMAR2 has a positive effect on the prognosis of patients with IBC, which may be related to the relatively high expression of its immune target genes.

The target genes in the prognostic model of this study included IL27RA, RAC2, FABP7, IGLV1-51, IGHA1, and IGHD, most of which have been reported in recent studies. IL27 is an immunomodulatory cytokine that can induce anti-tumour CD8 T cell responses in the tumour microenvironment. IL27 is also closely associated with mutant p53-driven tumourigenesis, and a lack of IL27 signalling (IL27RA- mice) significantly reduced mouse survival and increased the incidence of osteosarcoma (OS)in a previous study^25^. RAC2 promotes the occurrence and progression of OS by activating the Wnt signalling pathway. siRNA-RAC2 exhibits anti-tumour effects in vivo by inhibiting the proliferation and invasion of OS cells while facilitating OS cell apoptosis and impeding cell cycle progression^26^. Increased Wnt signalling is also thought to enhance the regulatory state of DCs by producing IL-10 and IL-12 in the tumour microenvironment, thereby promoting the antigen priming process of CD8 + T cells^27^. A previous study showed FABP7 as a potential target for the treatment of HER2 + breast cancer brain metastases. It is highly expressed in HER2 + breast cancer cells and is also necessary for the integrin-Src and vascular endothelial growth factor pathways. At the same time, in vitro experiments showed that FABP7 is crucial for the metabolic reprogramming of cancer cells^28^ (Fig. 6h). Miyauchi et al. found that IGHA1, a candidate gene for glioblastoma, was downregulated in the plasma of glioblastoma patients^29^. The immunoglobulin-related genes IGHA1 and IGHD have been reported to inhibit the recurrence of breast cancer^30^, which is consistent with the results of this study.

TMB and MSI have been reported as key biomarkers for the efficacy of immune checkpoint inhibitors (ICIs)^31,32^. In this study, we observed that the target immune genes of WAKMAR2 were highly expressed in a variety of tumours. WAKMAR2 was also found to be associated with TMB in half of the cancers studied, and with MSI in a small number of cancers. Immunotherapy has been acknowledged as a promising treatment option for IBC, but the response rate in IBC patients has remained relatively low^33^. It has been reported that high TMB is positively associated with the response rate of ICIs^34^. Our correlation analysis results demonstrated that patients with lower WAKMAR2 expression may have a poor response to ICIs. However, there is a strong correlation between WAKMAR2 and MSI in breast cancer. In addition, MSI has been reported to be a marker for PD-1 blockade^35^.

In summary, our results identified a potential molecular target for developing a novel immunotherapy for the treatment of breast cancer. Further research may optimise the screening of candidate patients in subsequent clinical trials and improve the development of individualised therapeutic schedules.

## Methods

### Identification of survival-related eRNAs and target genes in IBC

We obtained eRNA and its target gene sequences using an open-source, freely available tool, Human enhancer RNA Atlas (HeRA, https://hanlab.uth.edu/HeRA/) ^36^. The expression levels of eRNAs and the corresponding clinical information from 1104 IBC patients were obtained from The Cancer Genome Atlas (TCGA) database. We performed survival analysis to determine which eRNAs were related to IBC survival. We then performed a Pearson correlation analysis between these survival-related eRNAs and the target genes obtained from the HeRA website, using the screening conditions Cor > 0.4 and *P* < 0.01.

### Tumour microenvironment (TME) and single-sample gene set enrichment analysis (ssGSEA) of IBC patients

To observe the TME of patients with IBC, we applied an estimate algorithm based on the IBC gene set to calculate tumour purity and estimated scores for stromal cells and immune cells in malignant tumour tissues. We also obtained 29 immune-related gene sets from the literature and replaced these gene sets with gmt files^37^. Combined with the expression matrix of IBC samples, we used the "GSVA" package in R to perform single sample gene set enrichment analysis (ssGSEA) to obtain the expression of these 29 gene sets in different samples.

### Enrichment analysis of WAKMAR2-related immune target genes

We obtained the immune gene set from the website IMMPORT (https://www.immport.org/) and conducted Pearson correlation analysis with the eRNAs obtained above; the correlation coefficient was set to ≥ 0.4 or ≤  − 0.4 (*P* < 0.01). The regulatory network between eRNAs and immune target genes was constructed using Cytoscape (version 3.4.0), and the WAKMAR2 eRNA with the highest node degree in the network was screened. Subsequently, the "clusterProfiler" package in R was used to perform GO and KEGG^38^ enrichment analysis of the hub eRNA downstream immune genes. The enrichment results were visualised using chord diagrams and the pathways were considered noteworthy when the false discovery rate (FDR) was under 0.05.

### Prognosis model of WAKMAR2-related immune target genes

To obtain prognosis-related immune genes, univariate Cox analysis was performed using the target genes of WAKMAR2. Then, the prognosis model was constructed by applying the LASSO method, which screened prognostic predictors by keeping the regression coefficients less than the constant value. This prevented over-fitting of the model and, thus, more readily achieved clinical transformation. The LASSO method was applied using the R package glmnet. The risk score of each IBC patient in the prognostic model was calculated based on the prognostic characteristics using the following formula: risk score = expression of gene 1 × corresponding coefficient + expression of gene 2 × corresponding coefficient + expression of gene n × corresponding coefficient. Furthermore, the patients were divided into high- and low-risk groups by their median risk score, to validate the effectiveness of the model; in addition, the Kaplan–Meier analysis was applied to determine the difference in survival between the low- and high-risk groups. To evaluate the quality of the model, we performed a time-dependent ROC analysis to plot the AUC for 3, 5, and 10 years. We then combined clinical traits and risk scores to draw nomograms for each year. Subsequently, the Human Protein Atlas (https://www.proteinatlas.org/) was used to observe the immunohistochemical staining of these genes in normal and tumour breast tissues. Finally, the expression of WAKMAR2 and the results of estimate score and ssGSEA were visualised in the high- and low-risk groups using scatterplots, violin charts, and heatmaps, respectively, the relative fractions of immune cell subpopulations in different clusters were inferred using Xcell.

### Validation of WAKMAR2 and prognostic target genes in the pan-cancer landscape

To observe the potential role of WAKMAR2 in pan-cancer, we downloaded follow-up information and mutation data from 33 cancers in TCGA database and performed difference analysis and Kaplan–Meier analysis of WAKMAR2 in various cancers. MSI is closely related to the efficacy of immunosuppressive therapy and the reduction of tumour metastasis, and thus provides a good prognosis^32^. The TMB and MSI of each sample were calculated to analyse their correlation with WAKMAR2 expression. Oncomine (http://www.oncomine.org) was used to compare the expression of immune prognostic genes between tumours and healthy tissues.

### Molecular docking of WAKMAR2 and prognostic target genes

The secondary and tertiary structures of eRNAs and target genes were determined using the online programs RNAfold (http://rna.tbi.univie.ac.at/cgi-bin/RNAWebSuite/RNAfold.cgi) and RNAComposer (http://rnacomposer.ibch.poznan.pl/). The crystal structure of the transcription factor was obtained from the RCSB Protein Data Bank (https://www.rcsb.org/). The online tool HDock (http://hdock.phys.hust.edu.cn/) was used to predict and visualise nucleic acid-protein structures and nucleic acid-nucleic acid structures. TRANSFAC (http://gene-regulation.com/pub/databases.html) and position weight matrix (PWM) were used to search and screen for eRNA-related transcription factors.

### Patients

All methods were carried out in accordance with relevant guidelines and regulations. Ten patients who were diagnosed with IBC in the Department of Endocrinology and Breast Surgery of the First Affiliated Hospital of Chongqing Medical University, underwent a modified radical mastectomy, and samples of IBC and adjacent tissues were collected using standard procedures. The inclusion criteria of patients were as follows: (1) patients were pathologically diagnosed with triple-negative breast cancer; (2) patient tumours were classified as TNM stage I–II; and (3) the quality of each patient's immune function was checked 1 week before surgery. The protocol was approved by the Ethics Committee of the Affiliated Hospital of Chongqing Medical University (2020-135), and written informed consent was obtained from all patients.

### Cell culture and RNA interference

The human triple-negative breast cancer cell line MB-231 and MCF7 were purchased from ATCC. Cells were cultured in Dulbecco’s modified Eagle’s medium supplemented with 10% FBS, and maintained at 37 °C with 5% CO_2_. Experiments were performed during the early passages of cells. MB-231 and MCF7 cells were transfected with siRNA (25 nM) specific to WAKMAR2 (siRNA sequence1: tacatgcatacttctcttacact, siRNA sequence2: agcttaagttccgtgcattatct, siRNA sequence control: gacagaaacgtccaacaaactcc, synthesised by Ribobio, Guangzhou, China) using the Lipofectamine RNAiMAX Transfection Reagent (13778-150, ThermoFisher Scientific, IL, USA) according to the standard protocol.

### Total RNA extraction

The UNIQ-10 column total RNA extraction kit (Sangon Biotech) was used to extract total RNA from the IBC tissue. An SMA4000 microscope (Merington Instruments) and a DYY-6C electrophoresis instrument (Beijing Liuyi) were used to observe the concentration and quality of the RNA.

### Reverse transcription and qRT-PCR

The PrimeScript RT Reagent Kit (#RR047A, TaKaRa, China) was used to reverse transcribe total RNA, and the 2 × Fast SG qPCR Master Mix (High Rox, 3B639273, BBI Life Sciences) was used for qRT-PCR. GAPDH was used as an internal control. GraphPad Prism version 8.0 was used for the t-test. Primer sequences used were as follows:

- WAKMAR2 (forward primer, 5′-CTCTGCAGCAGTGACCTCAA-3′; reverse primer, 5′-AGTGGCAGGTATGAACGTGG-3′)
- IL27RA (forward primer, 5′-AAGTTCTGATCTGCCAGTTCCACTA-3′; reverse primer, 5′-GCTCCAAATCTTGGATCTCAAC-3′)
- RAC2 (forward primer, 5′-CGTCAGCCCAGCCTCTTATG-3′; reverse primer, 5′-TCAGGCCTCTCTGGGTGAG-3′)
- FABP7 (forward primer, 5′-TAAGTCTGTGGTTCGGTTGG-3′; reverse primer, 5′-CCCAAAGGTAAGAGTCACGAC-3′)
- GAPDH (forward primer, 5′-GCCCGTTTGCATTTTGTGGAGAG-3′; reverse primer, 5′-CCAACTTTCGGGAAATCCAT-3′).

### Western blot analysis

The cell lines were lysed with inhibitors contained RIPA buffer protease (Sigma-Aldrich). We used BCA protein assay kit (Promega) for protein quantification. Western blot analysis was performed as described previously^39^. The primary antibodies used in the western blot analysis were as follows: human anti- FABP7 (A11604, abclonal, 1:500), human anti‐IGHA1 (11449-1-AP, Sanying, 1:1000). We used Horseradish peroxidase-conjugated (HRP) goat anti-rabbit IgG (4050–05, SouthernBiotech, 1:20000) as a secondary antibody. Protein levels were normalised to GAPDH.

Chromatin immunoprecipitation (ChIP)MB231 and MCF7 cells were first cross-linked with 1% formaldehyde for 10 min. The cell pellets were then lysed, re-suspended, and subjected to sonication on ice. The samples were immunoprecipitated overnight at 4 °C on a rocking platform. Next, ChIP-grade Protein agarose beads were used to incubated with the lysate for 2 h at 4 °C^13^. The samples were washed with three different buffers and eluted in a buffer containing 5 M NaCl and 20 mg/mL Proteinase K. The input and immunoprecipitated samples were reverse cross-linked at 65 °C for 40 min. Finally, the elutes were used to detect ChIP signals by PCR.

## Supplementary Information

Supplementary Figure.

## Data Availability

The datasets used and/or analysed during the current study are available from the corresponding author on reasonable request.
