# Supplementary material for: A prospective study revealing the role of an immune-related eRNA, WAKMAR2, in breast cancer
Source: Sci Rep. 2021 Jul 28;11:15328. doi: 10.1038/s41598-021-94784-3 (PMC8319425; doi:10.1038/s41598-021-94784-3)
Supplement: Supplementary file 1 — Supplementary Figure. [file 41598_2021_94784_MOESM1_ESM.pdf]

SUPPLEMENTARY MATERIAL

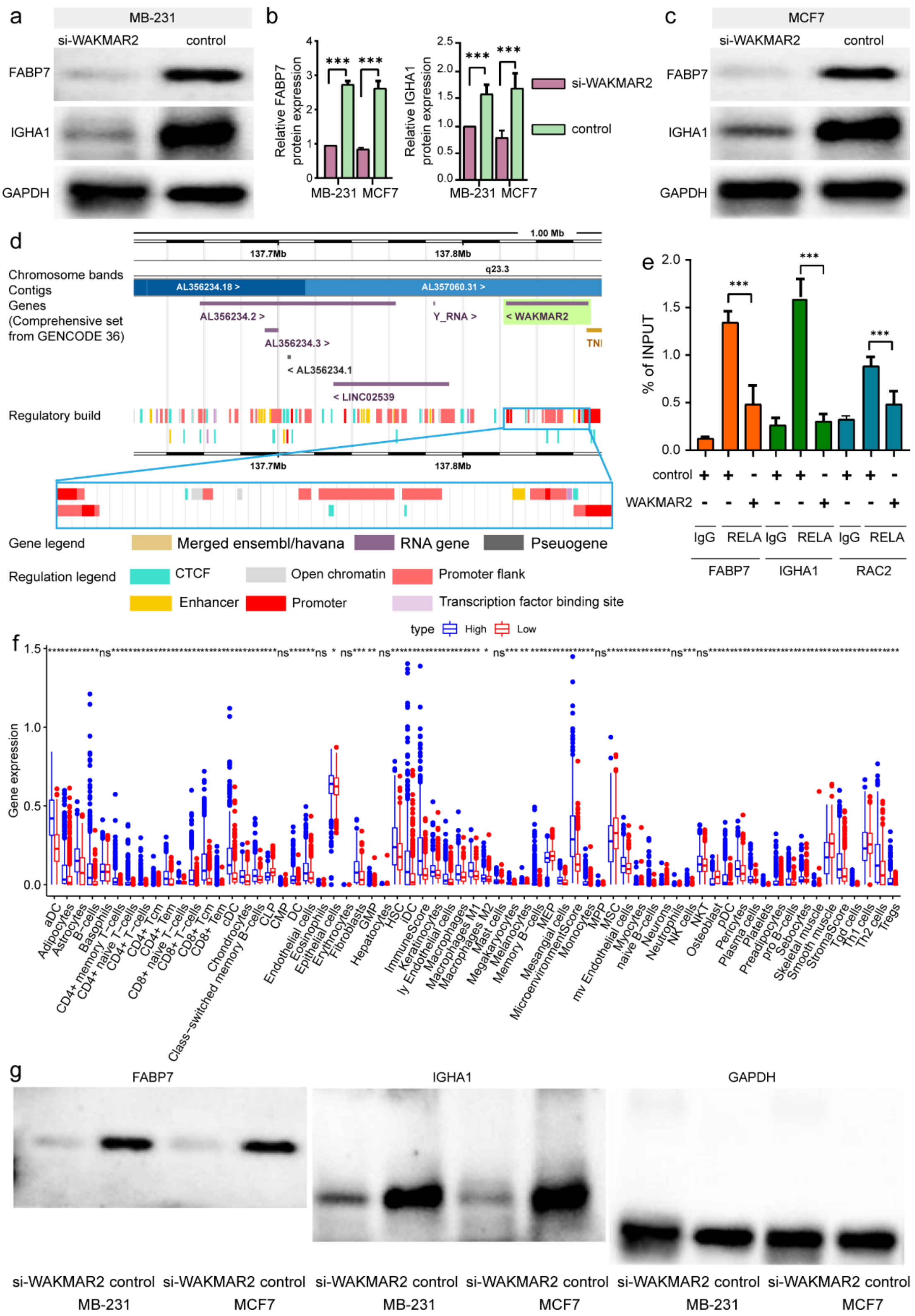


supplementary figure legend: (a-c) WB analysis of cell lines conducted with siRNA. (d) Identification of WAKMAR2 as eRNA by Ensembl. (e) ChIP experiment of eRNA and target genes. (f) Relative immune cell infiltration rate in WAKMAR2 high-expression and low-expression group. (g) Original, unprocessed versions of western blot analysis.
